# Presence of specific SARS-COV2 antibodies in hemodialysis patients and their caregivers after the first wave of COVID-19

**DOI:** 10.1038/s41598-022-15913-0

**Published:** 2022-07-13

**Authors:** Thomas Robert, Guillaume Lano, Noémie Resseguier, Mickaël Bobot, Dammar Bouchouareb, Stéphane Burtey, Xavier de Lamballerie, Jean Dhorne, Bertrand Dussol, Ariane Duval, Julien Faraut, Toscane Fourié, Philippe Giaime, Mourad Hallah, Dominique Jaubert, Océane Jéhel, Tristan Legris, Stéphane Liotatis, Valérie Moal, Laetitia Ninove, Nathalie Pedinielli, Marion Pelletier, Manon Romeu-Giannoli, Mariela Saba, Marion Sallée, Laurent Samson, Adriana Saveanu, Violaine Scarfoglière, Pascale Sebahoun, Romain Vial, Clarissa Von Kotze, Philippe Brunet, Gaëtan Lebrun, Stanislas Bataille, Noémie Jourde-Chiche

**Affiliations:** 1 AP-HM, CHU Conception, Centre de Néphrologie et Transplantation Rénale, 147 Bd Baille, 13005 Marseille, France; 2 MMG, Bioinformatics and Genetics, UMR_S910, Aix-Marseille Université, Marseille, France; 3 INSERM, INRAe, C2VN, Aix-Marseille Université, Marseille, France; 4 Laboratoire de Santé Publique, CERESS, Aix-Marseille Université, Marseille, France; 5 Unité des Virus Émergents, IRD 190, Inserm 1207, Aix-Marseille Université, Marseille, France; 6 AP-HM, Direction de la Recherche Santé, Marseille, France; 7 AP-HM, Centre d’Investigation Clinique, CHU la Conception, Marseille, France; 8 Association des Dialysés de Provence et Corse (ADPC), Marseille, France; 9 Institut Phocéen de Néphrologie, Clinique Bouchard, Marseille, France; 10 Service de Néphrologie et Hémodialyse, Centre Hospitalier Intercommunal Aix-Pertuis (CHIAP), Aix-en-Provence, France

**Keywords:** Renal replacement therapy, Epidemiology, Viral infection

## Abstract

Hemodialysis (HD) patients are at risk for severe COVID-19 and cannot comply with social distancing. SARS-COV2 seroprevalence in French patients and caregivers after the first wave of COVID-19 is unknown. SeroCOVIDial is a prospective study conducted between June and December 2020. SARS-COV2 seroprevalence was evaluated by a rapid serological test (BIOSYNEX) in HD patients and caregivers, and the presence or not of anti-SARS-COV2 neutralizing or non-neutralizing antibodies in patients was also determined by ELISA and seroneutralization. In June 2020, 451 HD patients and 238 caregivers were included. Overall SARS-COV2 seroprevalence was 8.4% (patients) and 6.7% (caregivers), and was 87.1% (patients) and 90.0% (caregivers) in participants with a previously documented SARS-COV2 infection. Overall seroprevalence reached 13.8% (patients) and 12.6% (caregivers) following the second epidemic wave. During the follow-up, 38 (8.4%) patients died (9 of COVID-19). Among the 44 (10.6%) patients who became infected, only two were seropositive at M0. The levels of anti-SARS-COV2 antibodies decreased over time in patients and caregivers. The BIOSYNEX test showed 82.9% sensitivity and 97.7% specificity. Prevalence of anti-SARS-COV2 antibodies was low in HD patients and caregivers after the first epidemic wave but rose after the second wave. A rapid serological test showed good performances and could be useful for future monitoring of anti-SARS-COV2 antibodies.

## Introduction

Patients on maintenance dialysis are a fragile population at high risk of severe coronavirus disease-2019 (COVID-19), with mortality rates reaching 20–30% in immunologically naïve patients^1–3^. Because they need to come to the dialysis facility for their dialysis sessions, usually three times a week, patients on dialysis are not able to comply with optimal social distancing, and are exposed to a risk of contamination by transporters, caregivers or other patients on dialysis. During the first epidemic wave in Europe, recommendations were issued on the systematic screening for symptoms in patients at each dialysis session, with dedicated isolated areas for positive patients^4^. As soon as vaccines against the severe acute respiratory syndrome coronavirus 2 (SARS-COV2) became available, patients on dialysis were considered a priority population for vaccination by international guidelines^5,6^. But few data is available on the spreading of the epidemic in this population and in their caregivers before the vaccination campaign.

Patients on dialysis are known to display altered response to some vaccines, and increased infectious risk compared to the general population^7^. Yet, preliminary reports showed a high occurrence of anti-SARS-COV2 antibodies after symptomatic COVID-19^8–11^ in patients on dialysis. Whether this presence of anti-SARS-COV2 antibodies persists over time, and is protective against a new SARS-COV2 infection, is still largely unknown. Moreover, the value of rapid serological tests to monitor patients for presence of anti-SARS-COV2 antibodies is unknown in this population.

The primary objective of this work was to determine the sero-prevalence and kinetics of anti-SARS-COV2 antibodies in a multicenter cohort of patients on dialysis and in their caregivers, after the first wave of COVID-19 and before the vaccination campaign. Secondary objectives were: (i) to evaluate the persistence of anti-SARS-COV2 antibodies, (ii) to evaluate the proportion of asymptomatic infections in patients and caregivers and determine if comorbid conditions were associated with more symptomatic infections, (iii) to evaluate the prevalence of anti-SARS-COV2 antibodies in participants with a documented infection before inclusion, (iv) to describe participants’ outcomes during the study period, (v) to determine the prevalence anti-SARS-COV2 neutralizing and non-neutralizing antibodies, assessed by laboratory tests, among dialysis patients, (vi) to evaluate the diagnostic performances of the rapid serological test BIOSYNEX in dialysis patients.

## Results

### Baseline characteristics of patients and caregivers

**Figure 1:**
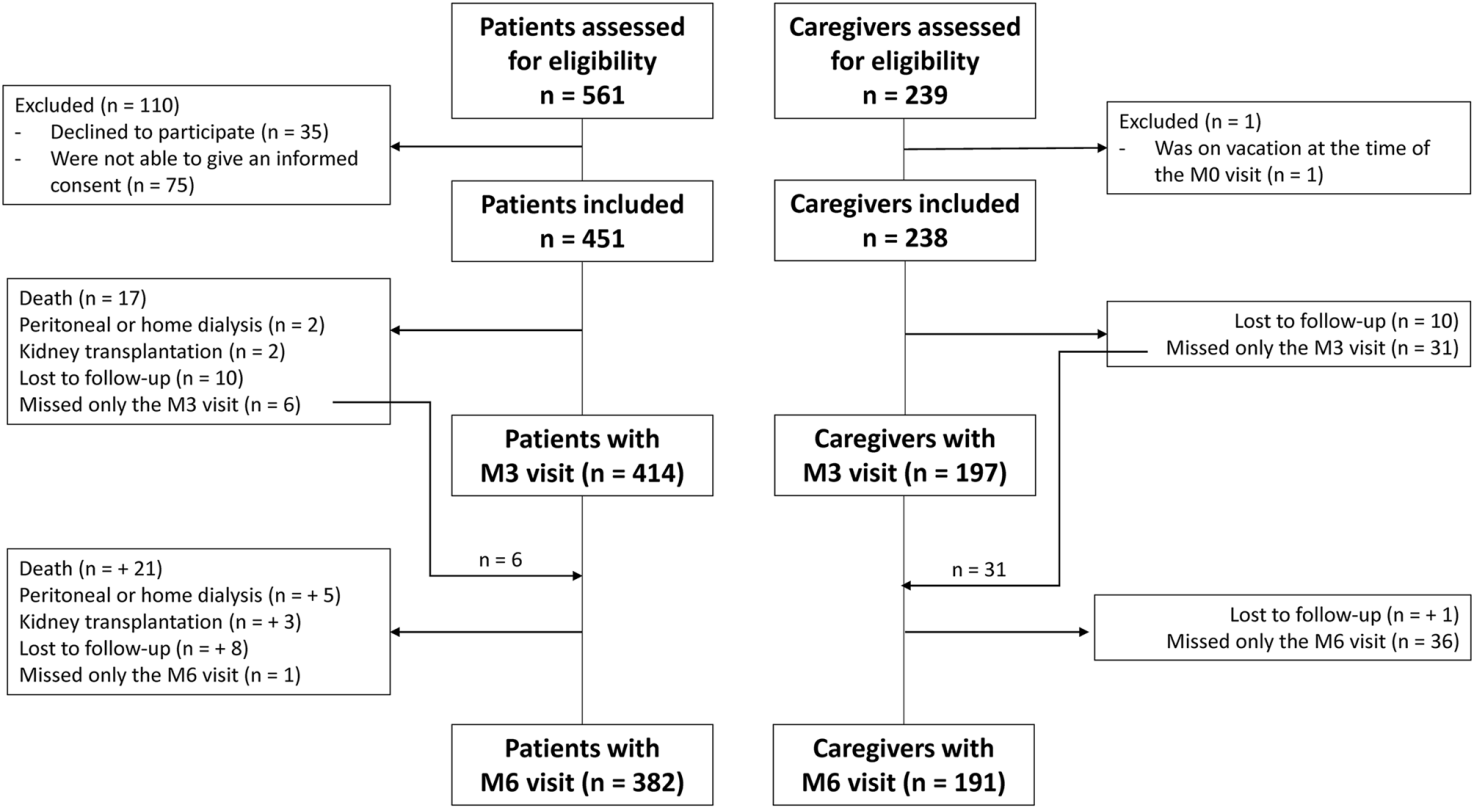
Flow diagram of participants (patients on the one hand, and caregivers on the other hand). Some participants, who missed the M3 visit, had the M6 visit.

In June 2020, 451 HD patients and 238 caregivers from 4 dialysis facilities of the Aix-Marseille area were included in the SeroCOVIDial study (Fig. 1). Baseline characteristics of patients and caregivers are presented in Table 1A, B. In HD patients, mean age was 66.5 years, and 57% of patients were male. The majority of patients displayed comorbidities such as hypertension or diabetes, 58% had presented cardio-vascular events, and 32% were unable to walk without help.

**Table 1:** Baseline characteristics of participants.

A. Patients	N = 451
Age, mean ± SD	66.5 ± 16.3
Age ≥ 65 years, n (%)	267 (59.2)
Gender Male, n (%)	259 (57.4)
Body mass index, mean ± SD	25.6 ± 5.4
Obesity, n (%) *Missing data*	84 (18.6) *19 (4.2)*
Smoking, n (%) *Missing data*	61 (13.5) *8 (1.8)*
Diabetes, n (%)	194 (43.0)
Hypertension, n (%)	354 (78.5)
Cardio-vascular disease, n (%)	262 (58.1)
Chronic respiratory disease, n (%)	102 (22.6)
Auto-immune disease, n (%)	27 (6.0)
Cancer, n (%)	57 (12.6)
Past history of kidney transplantation, n (%)	62 (13.75)
Listed on a kidney transplant waiting list, n (%)	81 (18.0)
Inability to walk without help, n (%)	146 (32.4)

B. Caregivers	N = 238
Age, mean ± SD	41.5 ± 11.7
Age ≥ 65 years, n (%)	1 (0.4)
Gender male, n (%)	57 (23.9)
Body mass index, mean ± SD	23.8 ± 4.4
Obesity, n (%) *Missing data*	18 (7.6) *10 (4.2)*
Smoking, n (%)	53 (22.3)
Diabetes, n (%)	4 (1.7)
Hypertension, n (%)	10 (4.2)
Cardio-vascular disease, n (%)	5 (2.1)
Chronic respiratory disease, n (%)	25 (10.5)
Auto-immune disease, n (%)	11 (4.6)
Cancer, n (%)	3 (1.3)

(A) Patients on dialysis. (B) Caregivers. Values are expressed as mean ± SD or n (%). Missing data are specified (given in italics).

### Prevalence of specific anti SARS-COV2 antibodies at inclusion and during the follow-up

The sero-prevalence at M0 (BIOSYNEX test) was 8.4% [95%CI, 6.0–11.4] in HD patients (Table 2), and 6.7% [95%CI, 3.9–10.7] in caregivers. This prevalence was not associated with comorbidities in patients or in caregivers (data not shown).

**Table 2:** Results of the serological tests and sero-neutralization test in hemodialysis patients. Results are expressed as number (% of samples tested).

	M0 N = 451	M3 N = 414	M6 N = 382
**Rapid serological test**			
**BIOSYNEX+ **	**38 (8.4)**	**28 (6.8)**	**52 (13.8)**
BIOSYNEX −	413 (91.6)	383 (93.2)	324 (86.2)
*Missing data*	*0*	*3*	*7*
**Main laboratory tests**			
**Anti-S ELISA + **	**43 (9.6)**	**32 (7.9)**	**62 (16.8)**
Anti-S ELISA −	405 (90.4)	375 (92.1)	307 (83.2)
*Missing data*	*3*	*7*	*13*
**Sero-neutralization + **	**26 (5.8)**	**27 (6.6)**	**59 (16.0)**
Sero-neutralization –	422 (94.2)	380 (93.4)	310 (84.0)
*Missing data*	*3*	*7*	*13*
**Immunization status**			
Anti-SARS-COV2 neutralizing antibodies	**26 (5.8)**	**27 (6.6)**	**59 (16.0)**
Anti-SARS-COV2 non-neutralizing antibodies	17 (3.8)	5 (1.2)	3 (0.8)
Absence of anti-SARS-COV2 antibodies	405 (90.4)	375 (92.2)	307 (83.2)
*Missing data*	*3*	*7*	*13*
**Additional laboratory tests**			
Anti-RBD ELISA +	37 (8.3)	32 (7.9)	51 (14.2)
Anti-RBD ELISA –	408 (91.7)	373 (92.1)	308 (85.8)
*Missing data*	*6*	*9*	*23*

Values with significant differences between M6 and M0 or M3 are in bold.

The number of missing data is specified (given in italics).

The sero-prevalence at M3 was 6.8% [95% CI, 4.6–9.7] in HD patients, and 5.6% [95%CI, 2.8–9.8] in caregivers. This was not significantly different from the sero-prevalence at M0 in patients or in caregivers.

The sero-prevalence at M6 was 13.8% [95% CI, 10.5–17.7] in HD patients, and 12.6% [95%CI, 8.2–18.1] in caregivers. The sero-prevalence was higher at M6 than at M0, both in patients (p = 0.003) and in caregivers (p = 0.007). It was also higher at M6 than at M3, both in patients (p = 0.0001) and in caregivers (p = 0.0003).

### Persistence of specific anti SARS-COV2 antibodies over time

Among the 38 patients with a positive BIOSYNEX test at M0, 25 were still tested positive at M3 (7 were tested negative, and 6 were not tested), and 18 were still tested positive at M6 (13 were tested negative, and 7 were not tested).

Among the 16 caregivers with a positive BIOSYNEX test at M0, 10 were still tested positive at M3 (4 were tested negative, and 2 were not tested), and 7 were still tested positive at M6 (4 were tested negative, and 5 were not tested).

### Proportion of asymptomatic infections

Among the 38 patients with a positive BIOSYNEX test at M0, 28 (73.7%) had presented symptoms of COVID-19, while 10 (26.3%) had reported no symptom since the beginning of the pandemic. The only comorbidity associated with the absence of symptoms was obesity: obese patients were more likely to have presented an asymptomatic infection than non-obese patients (p = 0.019) (data not shown).

Among the 16 caregivers with a positive BIOSYNEX test at M0, 15 (93.8%) had presented symptoms of COVID-19, while 1 (6.2%) reported no symptom. No characteristic or comorbidity was associated with asymptomatic infections in caregivers (data not shown).

### Sero-prevalence at M0 in participants with a documented SARS-COV2 infection before M0

Before M0, 31 patients from the cohort had presented a SARS-COV2 infection documented by RT-PCR on a nasopharyngeal swab. Among them, 27 (87.1%) had a positive BIOSYNEX test at M0. Among these patients with a positive test at M0, 21 were tested at M3 and M6: 20 (95%) and 16 (76%) were still tested positive at M3 and M6, respectively.

Similarly, 10 caregivers had presented a SARS-COV2 infection before M0, among whom 9 (90%) had a positive BIOSYNEX test at M0. Among these 9 caregivers, 7/9 (78%) and 5/7 (71%) were still tested positive at M3 and M6, respectively.

### Participants’ outcomes

During the 6-month follow-up, 38 (8.4%) patients died (17 patients between M0 and M3, and 21 additional patients between M3 and M6). Among them, 9 died of COVID-19 (none had a positive BIOSYNEX test at M0). No patient deceased of COVID-19 was obese. During the follow-up, 12 (2.7%) additional patients were hospitalized for COVID-19 (none had a positive BIOSYNEX test at M0).

Overall, among the 414 patients with a complete follow-up (follow-up until M6 or death), 44 (10.6%) presented a SARS-COV2 infection during the study period. Among these 44 patients, only 2 (4.5%) had a positive BIOSYNEX test at M0, and had no clinical severity of COVID-19.

Among the 191 caregivers with a complete follow-up, 14 (7.3%) presented a SARS-COV2 infection (none was hospitalized). None had a positive BIOSYNEX test at M0.

### Results of laboratory tests in hemodialysis patients

The results of laboratory tests and presence of anti-SARS-COV2 neutralizing and non-neutralizing antibodies in hemodialysis patients at M0, M3 and M6, are detailed in Table 2.

Among the 31 patients with a documented SARS-COV2 infection before M0, 24 (77.4%) had anti SARS-COV2 neutralizing antibodies, 2 (6.4%) had anti-SARS-COV2 non-neutralizing antibodies and 5 (16.1%) had no anti-SARS-COV2 antibodies at M0.

Among the 44 patients who presented a SARS-COV2 infection during the study period, 43 (97.8%) had no anti-SARS-COV2 antibodies at M0. One patient, who had anti-SARS-COV2 neutralizing antibodies at M0, was hospitalized two months later in the Intensive Care Unit for severe COVID-19. Of note, this patient had a negative BIOSYNEX test at M0. Symmetrically, the 2 patients who developed a SARS-COV2 infection despite a positive BIOSYNEX test at M0 had no anti-SARS-COV2 antibodies at M0.

The evolution of anti-S1 and anti-RBD titers, as well as the sero-neutralization titers over time in patients who had anti-SARS-COV2 antibodies at M0, are shown in Supplementary Fig. 1.

### Diagnostic accuracy of the rapid serological test

Diagnostic performances of the BIOSYNEX test were evaluated compared to the positivity of the anti-S1 ELISA and to the presence of anti-SARS-COV2 neutralizing antibodies, and are detailed in Table 3. Compared to the presence of anti-SARS-COV2 neutralizing antibodies, intrinsic properties of the BIOSYNEX test were 82.9% sensitivity and 97.7% specificity. The relation between pre-test and post-test probabilities, related to the likelihoods ratios of the BIOSYNEX test, are presented in Supplementary Fig. 2.

**Table 3:** Diagnostic performances of the BIOSYNEX test in hemodialysis patients.

BIOSYNEX compared to	Sensitivity	Specificity	Positive predictive value	Negative predictive value
Anti-S ELISA+	69.1 (60.6–96.8)	97.9 (96.8–98.7)	80.3 (72.0–87.1)	96.2 (94.9–97.2)
Anti-SARS-COV2 neutralizing antibodies	82.9(74.6–89.4)	97.7 (96.7–98.5)	78.6 (70.1–85.7)	98.3 (97.3–99.0)

All samples (M0 + M3 + M6) with both BIOSYNEX results and laboratory results were considered. Results are expressed as % (95% confidence interval).

## Discussion

In this large cohort of HD patients and their caregivers, we show that the prevalence of anti-SARS-COV2 antibodies after the first wave of COVID-19 in France was relatively low. It remained stable over the summer 2020, but increased in autumn as the second wave hit the country. A large majority of patients and caregivers who had a documented SARS-CoV-2 infection before M0 had anti-SARS-COV2 antibodies at inclusion, and the rapid serological test allowed the detection *a posteriori *of a few asymptomatic infections. During the study period, anti-SARS-COV2 antibodies could be lost both in patients and caregivers. Patients who had anti-SARS-COV2 antibodies at inclusion were not necessarily protected from COVID-19 during the follow-up, although the majority of patients who became infected had no anti-SARS-COV2 antibodies at M0. COVID-19 was not the main cause of death in HD patients during the study period.

In a study from the United Kingdom^14^ performed in May 2020 (also before the vaccination campaign), comprising 356 patients on dialysis, the systematic screening for anti-SARS-CoV-2 antibodies (anti-RBD and anti-NCP) yielded a sero-prevalence of 36%, which is four times higher than in the present cohort at the same period. A higher rate of asymptomatic infection (40%) was also reported in this study. Another survey performed in July 2020 in the USA on 12,932 patients on dialysis showed a global sero-prevalence of 6%, ranging from 1 to 24% according to the location in the USA, highlighting disparities in COVID-19 incidence^15^. Although the Aix-Marseille area was not spared by the first epidemic wave of COVID-19, the sero-prevalence (confirmed by laboratory tests in patients) observed here was low both in patients and in caregivers. This could reflect the efficacy of the preventive strategies implemented as early as February 2020 in the dialysis facilities participating in the present study. The sero-prevalence yet increased after the second epidemic wave, reflecting the high incidence in the general population in this region. Of note, although the mortality rate was high during the study period in hemodialysis patients (8.4% of the cohort over 6 months), it was mostly unrelated to COVID-19 (2% of dialysis patients died of COVID-19). While obesity is associated with a poor prognosis of COVID-19 in the general population^16^, it was not associated with severe forms of COVID-19 in HD patients in a previous work^1^, and was associated with asymptomatic forms in this study.

Several reports indicate that most individuals following SARS-CoV-2 infection develop immunoglobulin M (IgM), IgG, and IgA responses targeting the nucleocapsid (NCP) or the spike (S) protein of SARS-CoV-2, 7–14 days after infection^17–19^. The development of serological tests for SARS-CoV-2 antibodies has been an area of intense investigation^20,21^ and a number of tests are now commercially available. The rapid serological test used in the present study (BIOSYNEX) detects anti-S1 IgM and IgG antibodies, directed against the RBD domain. It demonstrated 96% sensitivity and 98% specificity when compared to anti-S1 ELISA in a panel from the general population^22^. Here, the BIOSYNEX test demonstrated lower sensitivity compared to anti-S1 ELISA in HD patients, but was more sensitive when compared to the presence of specific anti SARS-COV2 neutralizing antibodies. This immunization status requires the ability of the serum to inhibit viral proliferation in vitro (sero-neutralization). Although sero-neutralization is correlated with anti-S1 antibody titers^23^, it is a functional test that could predict effective clinical protection against SARS-CoV-2 better than mere serological tests ^24^. It has been proposed for the monitoring of anti-SARS-COV2 antibodies in immunocompromised patients, for the screening of patients who may benefit from a new vaccine injection, or from prophylactic anti-SARS-COV2 monoclonal antibody infusions in particular. Yet, it can be proposed only in highly specialized laboratory facilities. In this prospect, the good performances of the BIOSYNEX test compared to the presence of anti SARS-COV2 neutralizing antibodies is of particular interest.

Detailed description and precise estimates concerning the magnitude and duration of humoral responses to SARS-CoV-2 infection in patients on dialysis are lacking, as well as correlates of protective immunity following infection. In the general population, persistence of the SARS-CoV-2 anti-NCP antibodies response seems to be stable for at least 9 months. In patients on dialysis, anti-NCP antibodies remains detectable in only 36% of patients at 6 months while anti-RBD were still positive in 85% of patients ^10,25,26^. Anti-RBD antibody correlate more closely with neutralizing antibodies than anti-NCP antibody^27^. In the general population following a COVID-19 infection, neutralizing antibodies were developed in approximately 40% of anti-NCP antibody-positive individuals and was sustained for at least 9 months regardless of whether individuals were asymptomatic and suggest that most individuals remain susceptible to SARS-Cov-2 infection after a first infection^25^. Lower proportion of individuals with asymptomatic COVID-19 developed neutralizing antibodies compared to patient with symptomatic COVID-19^25^. Here, specific anti SARS-COV2 antibodies could be lost in caregivers, who reflect the general population, as well as in HD patients. Since the conduct of the present study, vaccination campaigns have been implemented in dialysis facilities. Most patients responded to 2 doses of ARN vaccines but not all of them had neutralizing antibodies^28^. This calls for a monitoring of the monitoring of anti-SARS-COV2 neutralizing antibodies against SARS-CoV-2 during the pandemic in this fragile population. This monitoring could be based on a rapid serological test such as BIOSYNEX, easily performed at the bedside on a drop of blood during the dialysis session.

This study has limitations. First, there was no systematic screening by PCR of SARS-CoV-2 infection in all patients and caregivers, but only a screening performed in case of symptoms or exposure. The real incidence of SARS-CoV-2 infection may thus be underestimated in this study, in particular in asymptomatic participants. Second, laboratory tests were not performed in caregivers, and the diagnostic performances of the BIOSYNEX test could not be evaluated in this population.

This study also has strengths. First, it was coordinated in time: the 689 participants were included over 2 weeks in June 2020, to ensure that their visits would take place at the same time of the epidemic waves. Second, a systematic screening for symptoms of COVID-19 was performed in the participating centers since the beginning of the pandemic and during all the study period. Third, ELISA and sero-neutralization assays were performed to determine the presence of anti SARS-COV2 neutralizing and non-neutralizing antibodies in hemodialysis patients and propose a validated gold standard to evaluate the diagnostic performances of the BIOSYNEX test.

To conclude, this study shows a low prevalence of anti-SARS-COV2 antibodies in hemodialysis patients and their caregivers after the first epidemic wave in France, which increased after the second wave. The large majority of patients who became infected during the follow-up had not anti-SARS-COV2 antibodies at M0, but a loss of anti-SARS-COV2 antibodies over time was observed both in patients and in caregivers. The rapid serological test had good performances when compared to the presence of anti-SARS-COV2 neutralizing antibodies, and could be useful for the monitoring of patients.

## Materials and methods

### Study design and participants

SeroCOVIDial is a prospective multicenter cohort study conducted over 6 months in patients on maintenance hemodialysis and in their caregivers, registered in clinicaltrials.gov (NCT04420338, first registration 09/06/2020). The study was conducted in accordance with the declaration of Helsinki, and was approved by the Comité de Protection des Personnes Ile-de-France II (approval 2020-AO1515-34). All participants gave their written informed consent before any study procedure.

All participants were included in a coordinated manner over 2 weeks in June 2020. Follow-up visits were performed in September 2020 (M3) and December 2020 (M6). Inclusion criteria for patients were: age ≥ 18 years, patient on maintenance hemodialysis, affiliated to the Social Security, able to give a written informed consent. Inclusion criteria for caregivers were: age ≥ 18 years, caregiver working with patients on maintenance hemodialysis, written informed consent.

The screening strategy for SARS-COV2 infection in patients was the same in the 4 dialysis facilities participating in this study, since the beginning of the pandemic in France, and throughout the study: patients were systematically questioned, at each dialysis session, about symptoms of COVID-19 or contact with infected persons; a nasopharyngeal swab for reverse transcription polymerase chain reaction (RT-PCR) SARS-COV2 test was performed (and repeated as needed) in all patients with symptoms and in contact cases. In caregivers, symptoms and potential diagnostic tests were collected at each visit by a self-administered questionnaire.

For the purpose of the study, all participants (patients and caregivers) had a rapid serological test (BIOSYNEX) on a drop of blood at M0, M3 and M6. A panel of serological and virological tests, performed at the Laboratory of Virology, was also performed on patients’ serum at M0, M3 and M6. Blood was drawn in patients at the beginning of a dialysis session.

### Definitions and outcomes

Presence of specific anti SARS-COV2 antibodies was defined as the detection of IgG, IgM, or IgG + IgM antibodies on the rapid serological test (BIOSYNEX COVID-19 BSS, Fribourg, Switzerland)^29^. This test detects antibodies directed against the receptor-binding domain (RBD) of the Spike protein (S1) of SARS-COV2.

In addition, based on the results of the laboratory panel performed in dialysis patients, the presence of anti-SARS-COV2 neutralizing antibodies was defined by positive anti-S1 antibodies (ELISA) and positive sero-neutralization test; the presence of anti-SARS-COV2 non-neutralizing antibodies was defined by positive anti-S1 antibodies (ELISA) with no sero-neutralization.

SARS-COV2 infection was defined at a given time by the detection of viral RNA on nasopharyngeal swab, whether the participant was symptomatic or not, or retrospectively by the discovery of specific anti SARS-COV2 antibodies. Severe SARS-COV2 infection was defined by the need for hospitalization or death. Death was considered due to COVID-19 in patients who died while they were hospitalized for COVID-19, or who died from secondary infections, malnutrition or decubitus complications in the 3 months following COVID-19.

### Laboratory tests and diagnostic accuracy of the rapid serological test

In patients on dialysis, the following panel of serological and virological tests were performed at the Laboratory of Virology on serum drawn at the same visit (M0, M3 and M6):

- anti-spike (S1 domain) IgG ELISA assay (Euroimmun, Lübeck, Germany),
- sero-neutralization assay (virus neutralization test VNT100), performed on all samples with detectable anti-S1 antibodies, as previously described ^12,13^.

Of note, anti-receptor binding domain (RBD) IgG ELISA assay (Access SARS-CoV-2 IgG II, Beckman, California, USA) was also performed in samples from dialysis patients.

Results of the ELISA assay were expressed in standardized units (binding antibody units (BAU) per mL) as recommended by the manufacturer. Result of the sero-neutralization assay were expressed as the highest dilutions of serum (1/20–1/160) allowing the reversal of the cytopathic effect of SARS-COV2 after 4 days on cultured cells. Sera with a titer > 1/40 were considered as sero-neutralizing.

Diagnostic performances of the rapid serological test BIOSYNEX were assessed in reference to the presence of anti-SARS-COV2 neutralizing antibodies and to anti-S1 ELISA assay.

### Statistical analysis

All analyses were performed in HD patients on one hand and in caregivers on the other hand.

The baseline characteristics were first described. Quantitative variables are presented as means (± SD), and categorical variables are presented as numbers (percentages).

The prevalence of anti-SARS-COV2 antibodies at inclusion was estimated with its 95% confidence interval. This prevalence was compared according to comorbidities (categorical variables) using chi-square test when appropriate (Fisher test otherwise). The prevalence was also estimated at each visit (M3, M6) with its 95% confidence interval. The prevalence was then compared (M0/M3; M0/M6; M3/M6) using McNemar’s test for paired proportions.

The prevalence of anti-SARS-COV2 antibodies at M0 was specifically estimated in participants who had a documented SARS-COV2 infection before M0.

The proportion of asymptomatic infections was estimated in participants who had anti-SARS-COV2 antibodies at M0. This proportion was compared according to comorbidities (categorical variables) using chi-square test when appropriate (Fisher test otherwise).

Outcomes over the follow-up (hospitalization and death) were described using numbers (percentages).

Diagnostic accuracy of the rapid serological test was assessed in comparison with the presence of anti-SARS-COV2 antibodies in hemodialysis patients. Sensitivity, specificity, positive and negative likelihood ratios, positive and negative predictive values were estimated with their 95% confidence intervals.

All analyses were performed using R software version 4.0.3 (R Foundation for Statistical Computing, Vienna, Austria). All tests were two-sided. Statistical significance was defined as p < 0.05.

## Supplementary Information

Supplementary Figures.

## References

[1] Lano G, Braconnier A, Bataille S, Cavaille G, Moussi-Frances J, Gondouin B (2020). Risk factors for severity of COVID-19 in chronic dialysis patients from a multicentre French cohort. Clin. Kidney J..

[2] Jager KJ, Kramer A, Chesnaye NC, Couchoud C, Sánchez-Álvarez JE, Garneata L (2020). Results from the ERA-EDTA Registry indicate a high mortality due to COVID-19 in dialysis patients and kidney transplant recipients across Europe. Kidney Int..

[3] Hilbrands LB, Duivenvoorden R, Vart P, Franssen CFM, Hemmelder MH, Jager KJ (2020). COVID-19-related mortality in kidney transplant and dialysis patients: Results of the ERACODA collaboration. Nephrol. Dial. Transplant.

[4] Basile C, Combe C, Pizzarelli F, Covic A, Davenport A, Kanbay M (2020). Recommendations for the prevention, mitigation and containment of the emerging SARS-CoV-2 (COVID-19) pandemic in haemodialysis centres. Nephrol. Dial. Transplant..

[5] Council E-E, Group EW (2021). Chronic kidney disease is a key risk factor for severe COVID-19: A call to action by the ERA-EDTA. Nephrol. Dial. Transplant.

[6] Francis A, Baigent C, Ikizler TA, Cockwell P, Jha V (2021). The urgent need to vaccinate dialysis patients against severe acute respiratory syndrome coronavirus 2: A call to action. Kidney Int..

[7] Kato S, Chmielewski M, Honda H, Pecoits-Filho R, Matsuo S, Yuzawa Y (2008). Aspects of immune dysfunction in end-stage renal disease. Clin. J. Am. Soc. Nephrol..

[8] De Vriese AS, Reynders M (2020). IgG antibody response to SARS-CoV-2 infection and viral RNA persistence in patients on maintenance hemodialysis. Am. J. Kidney Dis..

[9] Shaikh A, Zeldis E, Campbell KN, Chan L (2021). Prolonged SARS-CoV-2 viral RNA shedding and IgG antibody response to SARS-CoV-2 in patients on hemodialysis. Clin. J. Am. Soc. Nephrol..

[10] Sakhi H, Dahmane D, Attias P, Kofman T, Bouvier M, Lapidus N (2021). Kinetics of anti-SARS-CoV-2 IgG antibodies in hemodialysis patients six months after infection. J. Am. Soc. Nephrol..

[11] Giot M, Robert T, Brunet P, Resseguier N, Lano G (2021). Vaccination against COVID-19 in a haemodialysis centre: What is the risk of bleeding complications?. Clin. Kidney J..

[12] Gallian P, Pastorino B, Morel P, Chiaroni J, Ninove L, de Lamballerie X (2020). Lower prevalence of antibodies neutralizing SARS-CoV-2 in group O French blood donors. Antiviral Res..

[13] Nurtop E, Villarroel PMS, Pastorino B, Ninove L, Drexler JF, Roca Y (2018). Combination of ELISA screening and seroneutralisation tests to expedite Zika virus seroprevalence studies. Virol. J..

[14] Clarke C, Prendecki M, Dhutia A, Ali MA, Sajjad H, Shivakumar O (2020). High prevalence of asymptomatic COVID-19 infection in hemodialysis patients detected using serologic screening. J. Am. Soc. Nephrol..

[15] Walker AG, Sibbel S, Wade C, Moulton N, Marlowe G, Young A (2021). SARS-CoV-2 antibody seroprevalence among maintenance dialysis patients in the United States. Kidney Med..

[16] Gao M, Piernas C, Astbury NM, Hippisley-Cox J, O'Rahilly S, Aveyard P (2021). Associations between body-mass index and COVID-19 severity in 6·9 million people in England: A prospective, community-based, cohort study. Lancet Diabetes Endocrinol..

[17] Shu H, Wang S, Ruan S, Wang Y, Zhang J, Yuan Y (2020). Dynamic changes of antibodies to SARS-CoV-2 in COVID-19 patients at early stage of outbreak. Virol. Sin..

[18] Guo L, Ren L, Yang S, Xiao M, Yang F (2020). Profiling early humoral response to diagnose novel coronavirus disease (COVID-19). Clin. Infect. Dis..

[19] Rongqing Z, Li M, Song H, Chen J, Ren W, Feng Y (2020). Early detection of severe acute respiratory syndrome coronavirus 2 antibodies as a serologic marker of infection in patients with coronavirus disease 2019. Clin. Infect. Dis..

[20] Prendecki M, Clarke C, McKinnon T, Lightstone L, Pickering MC, Thomas DC (2021). SARS-CoV-2 antibody point-of-care testing in dialysis and kidney transplant patients with COVID-19. Kidney Med..

[21] Moshe M, Daunt A, Flower B, Simmons B, Brown JC, Frise R (2021). SARS-CoV-2 lateral flow assays for possible use in national covid-19 seroprevalence surveys (React 2): Diagnostic accuracy study. BMJ.

[22] Péré H, MboumbaBouassa RS, Tonen-Wolyec S, Podglajen I, Veyer D, Bélec L (2021). Analytical performances of five SARS-CoV-2 whole-blood finger-stick IgG-IgM combined antibody rapid tests. J. Virol. Methods.

[23] Legros V, Denolly S, Vogrig M, Boson B, Siret E, Rigaill J (2021). A longitudinal study of SARS-CoV-2-infected patients reveals a high correlation between neutralizing antibodies and COVID-19 severity. Cell Mol. Immunol..

[24] Khoury DS, Cromer D, Reynaldi A, Schlub TE, Wheatley AK, Juno JA (2021). Neutralizing antibody levels are highly predictive of immune protection from symptomatic SARS-CoV-2 infection. Nat. Med..

[25] He Z, Ren L, Yang J, Guo L, Feng L, Ma C (2021). Seroprevalence and humoral immune durability of anti-SARS-CoV-2 antibodies in Wuhan, China: A longitudinal, population-level, cross-sectional study. Lancet.

[26] Clarke CL, Prendecki M, Dhutia A, Gan J, Edwards C, Prout V (2021). Longevity of SARS-CoV-2 immune responses in hemodialysis patients and protection against reinfection. Kidney Int..

[27] Premkumar L, Segovia-Chumbez B, Jadi R, Martinez DR, Raut R, Markmann A (2020). The receptor binding domain of the viral spike protein is an immunodominant and highly specific target of antibodies in SARS-CoV-2 patients. Sci. Immunol..

[28] Giot M, Fourié T, Lano G, Villarroel PMS, de Lamballeri X, Gully M (2021). Spike and neutralizing antibodies response to COVID-19 vaccination in hemodialysis patients. Clin. Kidney J..

[29] Péré H, MboumbaBouassa RS, Tonen-Wolyec S, Podglajen I, Veyer D, Bélec L (2021). Analytical performances of five SARS-CoV-2 whole-blood finger-stick IgG-IgM combined antibody rapid tests. J. Virol. Methods.

