# Supplementary material for: Presence of specific SARS-COV2 antibodies in hemodialysis patients and their caregivers after the first wave of COVID-19
Source: Sci Rep. 2022 Jul 13;12:11972. doi: 10.1038/s41598-022-15913-0 (PMC9279503; doi:10.1038/s41598-022-15913-0)
Supplement: Supplementary file 1 — Supplementary Figures. [file 41598_2022_15913_MOESM1_ESM.pdf]

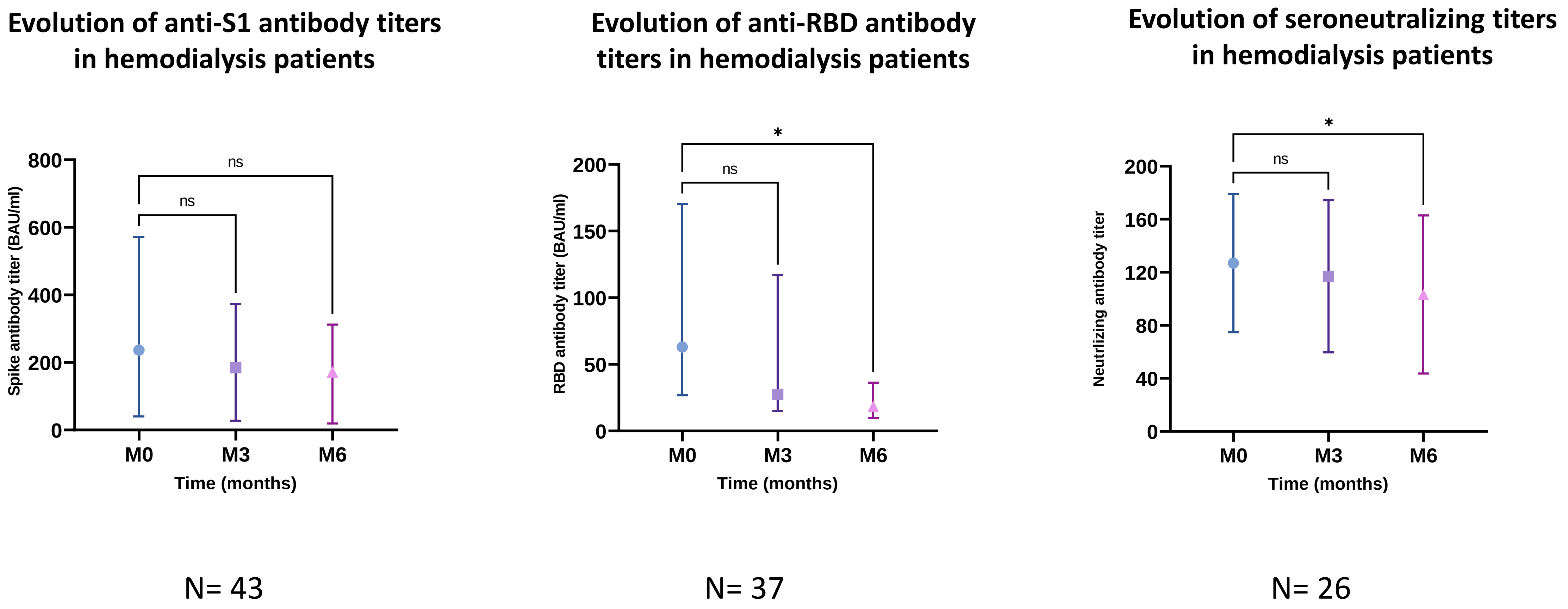

Supplementary Figure 1. Evolution of anti-SARS-COV2 antibody titers, and of sero-neutralizing titers, in dialysis patients immunized at inclusion. A. Evolution of anti-S1 antibody titers in patients with positive anti-S1 antibodies at M0. **B.** Evolution of anti-RBD antibody titers in patients with positive anti-RBD antibodies at M0. **C.** Evolution of sero-neutralization titers in patients with positive sero-neutralization test at inclusion.

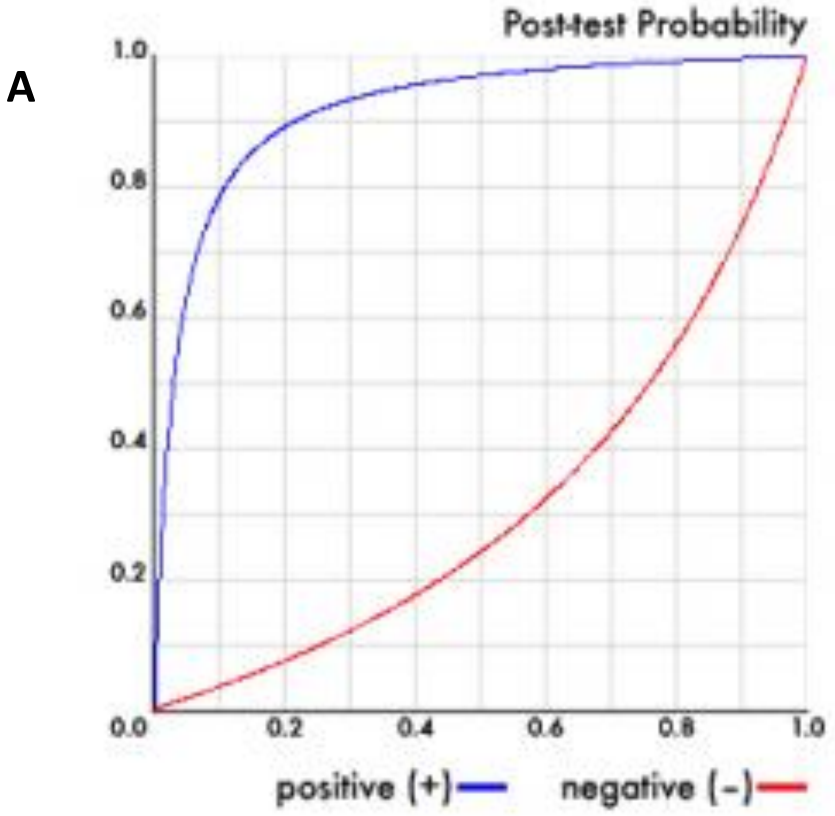

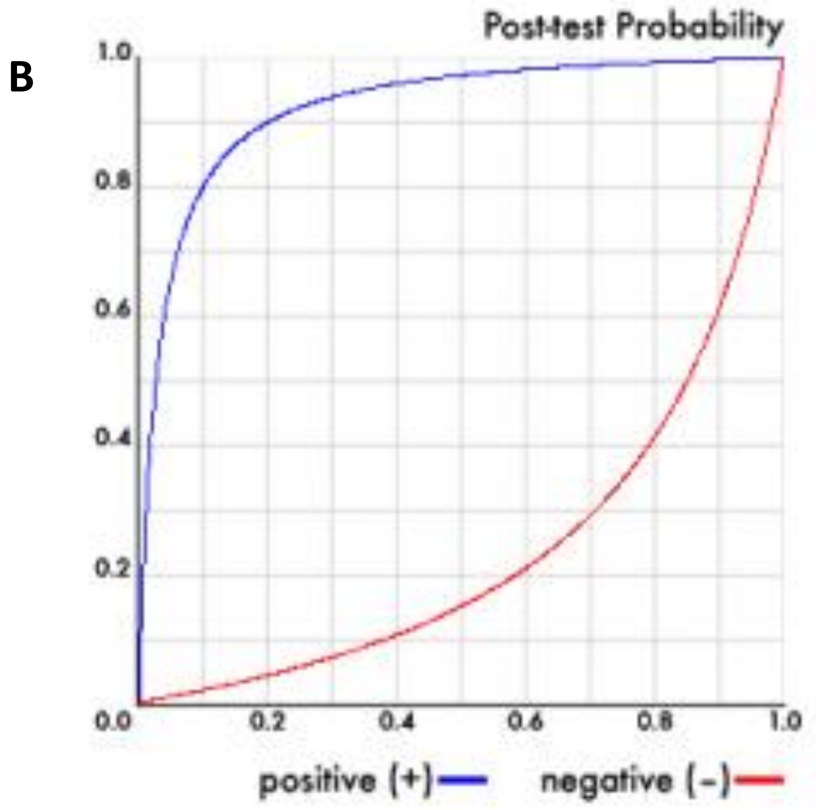

Supplementary Figure 2. Representation of the intrinsic value of the Biosynex® test, based on likelihood ratios, compared to anti-S1 ELISA and to the immunization status. The curves represent the post-test probability of having a positive (in blue) or negative (in red) anti-S1 ELISA assay **(A)** or full-immunization status **(B)**, according to the pre-test probability (x-axis) in a given population. Graphics were performed using the evidence-based medicine toolbox from https://ebm-tools.knowledgetranslation.net/calculator/diagnostic/.
